# Hypoxia sensing in the body: An update on the peripheral and central mechanisms

**DOI:** 10.1113/EP091206

**Published:** 2023-11-30

**Authors:** Daniel B. Zoccal, Beatriz N. Vieira, Letícia R. Mendes, Andressa B. Evangelista, Isabela P. Leirão

**Affiliations:** ^1^ Department of Physiology and Pathology, School of Dentistry of Araraquara São Paulo State University (UNESP) Araraquara São Paulo Brazil

**Keywords:** brainstem, cardiovascular system, carotid body, oxygen, spinal cord, sympathetic, ventilation

## Abstract

An adequate supply of O_2_ is essential for the maintenance of cellular activity. Systemic or local hypoxia can be experienced during decreased O_2_ availability or associated with diseases, or a combination of both. Exposure to hypoxia triggers adjustments in multiple physiological systems in the body to generate appropriate homeostatic responses. However, with significant reductions in the arterial partial pressure of O_2_, hypoxia can be life‐threatening and cause maladaptive changes or cell damage and death. To mitigate the impact of limited O_2_ availability on cellular activity, O_2_ chemoreceptors rapidly detect and respond to reductions in the arterial partial pressure of O_2_, triggering orchestrated responses of increased ventilation and cardiac output, blood flow redistribution and metabolic adjustments. In mammals, the peripheral chemoreceptors of the carotid body are considered to be the main hypoxic sensors and the primary source of excitatory feedback driving respiratory, cardiovascular and autonomic responses. However, current evidence indicates that the CNS contains specialized brainstem and spinal cord regions that can also sense hypoxia and stimulate brain networks independently of the carotid body inputs. In this manuscript, we review the discoveries about the functioning of the O_2_ chemoreceptors and their contribution to the monitoring of O_2_ levels in the blood and brain parenchyma and mounting cardiorespiratory responses to maintain O_2_ homeostasis. We also discuss the implications of the chemoreflex‐related mechanisms in paediatric and adult pathologies.

## Abstract (highlights)

- **What is the topic of this review?**
  Hypoxia is a life‐threatening condition. Specialized sensory mechanisms in the periphery and CNS respond to reduced O_2_ levels, triggering ventilatory and haemodynamic responses to enhance O_2_ uptake and delivery to the tissues.
- **What advances does it highlight?**
  Although anatomically distinct, both peripheral and central mechanisms should be considered when examining the homeostatic responses to hypoxia. Herein, we review past and current research on O_2_ monitoring in the periphery and CNS, discussing how their sensing mechanisms, pathways and interactions modify respiratory and sympathetic activities.

## 1 INTRODUCTION

Oxygen is fundamental for the survival and functioning of most forms of life on Earth. In air‐breathing animals, O_2_ is extracted from the atmospheric air and delivered to the cells for energy‐producing processes and biochemical reactions. A failure to sustain the physiological levels of O_2_ can have short‐ and long‐term detrimental consequences for cellular activity, especially in tissues with high vulnerability because of elevated metabolic demand and the absence of energy reserves, such as the CNS (Bailey, 2019). An inadequate supply of O_2_ to the tissues (or hypoxia) can occur owing to decreased O_2_ availability in the air (e.g., at high altitudes), in association with diseases (e.g., central apnoeas, airway obstruction, lung diseases and blood flow restriction), or by a combination of both. At the cellular level, enzymes exhibiting low O_2_ affinity (e.g., proline hydroxylase domain proteins controlling the stability of hypoxia‐inducible factors) can sense hypoxia and initiate biochemical and transcriptional responses or signalling pathway adaptations. However, integrated homeostatic and behavioural responses are essential to mitigate the effects of reduced O_2_ levels and support crucial body functions during exposure to hypoxia. Cells equipped with specialized sensory mechanisms that respond rapidly to moderate reductions in O_2_ levels in the body are responsible for initiating a cascade of systemic adjustments to hypoxia. These O_2_‐sensitive systems can be found in multiple vertebrate species, from water‐ to air‐breathing animals, suggesting that these mechanisms might be conserved during evolution. In mammals, O_2_ chemoreceptor cells providing excitatory feedback inputs to networks that control ventilation, cardiovascular function and metabolism are found in the vascular system and brain parenchyma. The functioning of these O_2_ chemoreceptors has been studied extensively; however, interrogations regarding their detailed operation, integration and plasticity indicate the need for further studies.

Herein, we review and discuss the mechanisms associated with chemotransduction in peripheral and central sensors, their interactions with respiratory and autonomic networks, and their contribution to modifying the breathing pattern and sympathetic outflow in health and disease.

## 2 OXYGEN HOMEOSTASIS AND THE RESPIRATORY AND AUTONOMIC ADJUSTMENTS DURING HYPOXIA

The maintenance of O_2_ levels in the blood and tissues depends on the coordinated activity of the cardiovascular and respiratory systems, regulating gas exchange, blood circulation and O_2_ diffusion between the compartments of the body. The frequency and depth of breathing determine the partial pressure of O_2_ in the alveolar space and its diffusion rate to the bloodstream. A collection of excitatory and inhibitory neurons in the ventral surface of the medulla oblongata forms the so‐called pre‐Bötzinger complex (preBötC), which generates intrinsic oscillations that determine the fundamental respiratory rhythm and drive breathing activity (Smith et al., 1991). The oscillatory activity of the preBötC is relayed to pontomedullary premotor and motor neurons that control the pattern (timing, shape and amplitude) of the upper airway, thoracic and abdominal muscles (Richter & Smith, 2014). Entrained with the respiratory system, the cardiovascular system supplies the alveoli with venous blood and distributes the oxygen‐enriched arterial blood to all tissues in the body. The autonomic nervous system, mainly through the sympathetic nerves controlling vascular diameter and heart activity, can rapidly modify arterial pressure levels and blood flow. The sympathetic vasoconstrictor drive is determined mainly by the activity of a cluster of excitatory neurons in the rostral ventrolateral medulla (RVLM), which is considered a major source of excitatory inputs to the preganglionic sympathetic neurons (Ross et al., 1984). These neurons receive synaptic projections from multiple brainstem and forebrain regions, including the respiratory neurons of the preBötC, indicating that this region integrates sympathetic control with other physiological systems (Guyenet, 2006). The sympathetic nervous system also regulates the energy expenditure and O_2_ demand of the body by controlling peripheral (e.g., cutaneous) blood flow and heat exchange with the environment, regulating non‐shivering thermogenesis through innervations to the brown adipose tissue and stimulating lipolysis and glycogenolysis (Madden & Morrison, 2005; Zsombok et al., 2023).

During brief exposure to hypoxia (seconds to a few minutes), the respiratory network is stimulated to elicit an immediate increase in pulmonary ventilation proportional to the decrease in the partial pressure of oxygen in the arterial blood ($P_{aO_{2}}$). This hypoxic ventilatory response (HVR) results from augmented motor activity to inspiratory muscles, such as the diaphragm, external intercostals and dilator upper airway muscles, causing increases in breathing frequency and tidal volume (Morris et al., 1996). Moreover, expiration becomes an active process, with the recruitment of abdominal and internal intercostal muscles to enhance the expiratory flow, further increasing alveolar ventilation and O_2_ uptake (Lemes & Zoccal, 2014). These hypoxia‐elicited adjustments in the breathing pattern rely on the activation of multiple areas of the brainstem respiratory network, including the preBötC, lateral parafacial group, hypoglossal nucleus, retrotrapezoid nucleus and pontine Kölliker–Fuse nucleus (Alheid et al., 2011; Biancardi et al., 2021; Song et al., 2011; Zoccal et al., 2018).

In association with the HVR, a rapid increase in the sympathetic activity to blood vessels, the heart and adrenal glands is observed during exposure to hypoxia that promotes: (1) vasoconstriction in muscular, cutaneous, renal and mesenteric beds; (2) vasodilatation in cardiac and cerebral circulations; (3) increases in ventricular stroke volume; and (4) release of noradrenaline in the circulation (Moraes, Machado, et al., 2014; Zera et al., 2019). This stereotyped sympatho‐excitatory response is believed to redistribute blood flow and O_2_ supply to sensitive organs, especially the brain, during hypoxic conditions. Alterations in the rhythmicity of the sympathetic discharge are also noticed during hypoxia, with the presence of noticeable respiratory‐modulated bursts in the sympathetic outflow (Moraes, Machado, et al., 2014). This strengthened respiratory–sympathetic coupling might represent a mechanism to entrain the HVR and sympatho‐excitation to optimize blood delivery to the lungs and tissues and improve blood gas exchange and perfusion. The sympatho‐excitatory response to hypoxia relies on the activation of presympathetic RVLM neurons in the ventral medulla (Koshiya & Guyenet, 1996); however, other forebrain and brainstem regions, such as the paraventricular nucleus of the hypothalamus and the A5 region in the pons, are also recruited and contribute to the adjustments in the sympathetic outflow (Reddy et al., 2005; Taxini et al., 2017).

If the hypoxic stimulus persists for several minutes to hours, the initial acute ventilatory response is followed by a secondary depression, whereby ventilation reaches a lower steady state (Powell et al., 1998). This secondary decline in ventilation is attributed to hypoxia‐induced reduced neuronal excitability (Martin et al., 2007). A decrease in the metabolic rate owing to suppression of thermogenesis also contributes to determining the level of HVR during the second phase (Saiki et al., 1994). There is no evidence of whether the sympathetic activity to the cardiovascular system undergoes a two‐phase pattern like the HVR. A fall in blood pressure levels can be observed during systemic hypoxia; however, it is secondary to the recruitment of myogenic autoregulatory mechanisms that cause vasodilatation at the tissue level (Bryan & Marshall, 1999) rather than a depression in the sympathetic vasoconstrictor outflow. However, exposure to hypoxia suppresses the activity of presympathetic neurons in the raphe pallidus that control sympathetic activity to brown adipose tissue (Madden & Morrison, 2005), contributing to the reduction in body temperature and metabolic rate.

If hypoxia persists for several hours to days, long‐lasting adaptations can be observed in the respiratory system, cardiovascular system and CNS. For example, in chronic conditions of sustained hypoxia, resting ventilation shows a progressive increase, a phenomenon named ventilatory acclimatization to hypoxia, which can persist after the return to normoxic conditions (Flor et al., 2018). There is also evidence for increases in baseline sympathetic discharge during sustained exposure to hypoxia, which might follow the development of the ventilatory acclimatization to hypoxia (Moraes, Bonagamba, et al., 2014). It is also described that sustained hypoxia amplifies the HVR and sympatho‐excitation and suppresses the hypothermic response during a new acute hypoxia challenge (Flor et al., 2018). The mechanisms underpinning cardiorespiratory adjustments during sustained hypoxia are complex and involve plasticity in peripheral and central systems regulating the respiratory and autonomic network functioning (Bisgard, 2000; Moraes, Bonagamba, et al., 2014).

## 3 PERIPHERAL O_2_ CHEMORECEPTORS

**FIGURE 1:**
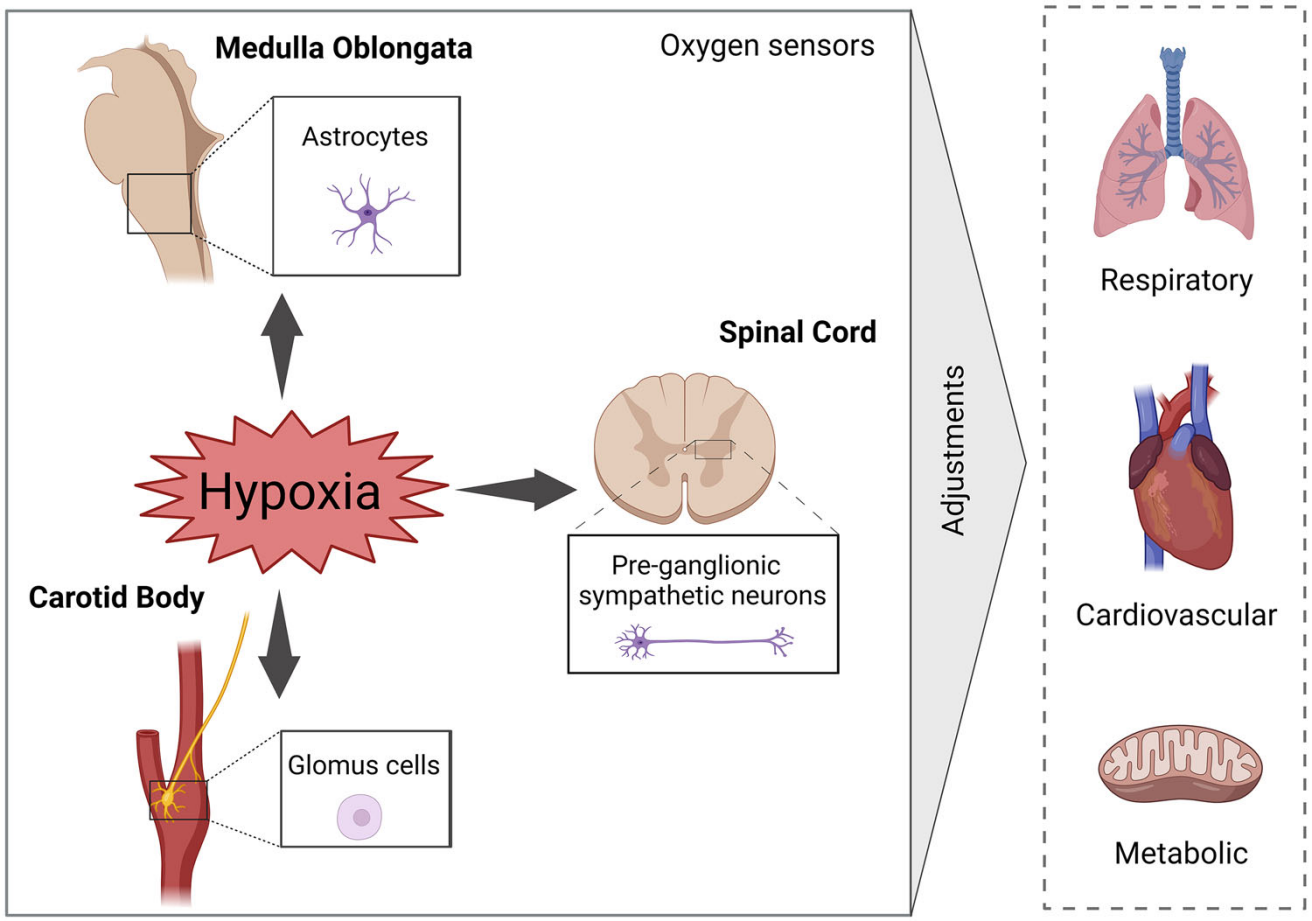
Hypoxia sensing in the body and the systemic compensatory responses. Schematic drawing illustrating the mammalian O_2_‐sensitive sites in the periphery and CNS. Cells that are excited during hypoxia have been characterized in the carotid bodies (glomus cells), brainstem (astrocytes in the ventrolateral medulla oblongata) and spinal cord (preganglionic sympathetic neurons in the intermediolateral column). Stimulation of these cells during low O_2_ availability triggers adjustments in brain networks controlling the respiratory and autonomic activities that, in turn, bring about ventilatory, cardiovascular and metabolic compensatory responses. Figure created with BioRender.

The identification of reductions in $P_{aO_{2}}$ is vital to trigger fast compensatory responses during exposure to hypoxia and to avoid tissue and cellular damage. Chemosensitive cells connected to the arterial system (Figure 1) are suggested as the primary responders that initiate systemic cardiorespiratory responses during hypoxia exposure (Mouradian et al., 2012). These cells are located bilaterally in the carotid bifurcation, in the carotid bodies (CBs), and were first recognized as a peripheral O_2_ sensor in the 1930s (Heymans & Bouckaert, 1930). The CBs contain blood vessels, afferent and efferent nerve terminals, and two clusters of cells. One group, named type I or glomus cells, is formed by dopaminergic cells in close contact with capillaries and afferent fibres. The glomus cells are the sensing element of the CB, containing O_2_‐sensitive K^+^ channels (Lopez‐Barneo et al., 1988). The other group of cells, named type II, are non‐excitable cells with a structural role, serving as precursors of new glomus cells and secreting transmitters that modulate the afferent output (Murali & Nurse, 2016). Currently, evidence shows that the CBs are a polymodal sensory organ, exhibiting sensing properties and evoking reflex responses to other stimuli, such as high carbon dioxide/acidosis, salt overload, low‐glucose and increased plasma concentration of insulin, leptin, angiotensin II and pro‐inflammatory molecules (Baby et al., 2023; da Silva et al., 2019; Katayama et al., 2022; Lahiri et al., 1978; Melo et al., 2020; Pardal & Lopez‐Barneo, 2002; Shin et al., 2019).

The mechanisms mediating the chemotransduction of hypoxia in the glomus cells have been studied extensively and are still a matter of investigation and discussion. Mitochondrion‐dependent signalling pathways contribute crucially to this sensory process. It is reported that O_2_ sensing by the glomus cell requires a functional mitochondrial complex I, which is inhibited during hypoxia, leading to incomplete electron transfer in the electron transport chain and accumulation of NADH and mitochondrial formation of reactive oxygen species (ROS) (Arias‐Mayenco et al., 2018). The suppression of the electron transport chain during hypoxia might also occur in complex IV, owing to its high O_2_ sensitivity produced by the association with proteins (HIGD1C and COX4I2) expressed almost exclusively in the glomus cells (Timon‐Gomez et al., 2022). Other studies propose the involvement of gaseous transmitters, such as the endogenously produced hydrogen sulphide (H_2_S), whose concentration increases in the glomus cells during hypoxia owing to suppressed mitochondrial oxidation (Peng et al., 2010). The increased intracellular concentration of these molecules in the glomus cells promotes the inhibition of K^+^ currents, membrane depolarization and a Ca^2+^‐dependent release of neurotransmitters (Telezhkin et al., 2010; Timon‐Gomez et al., 2022). A multitude of excitatory and inhibitory transmitters and modulators have been identified in the glomus cells, including ATP, dopamine, acetylcholine, histamine and serotonin (Prabhakar, 2006). However, the physiological significance of multiple neurotransmitters in the glomus cells remains unclear, especially for sensing hypoxia. It is known that some of these transmitters, such as ATP, bind to postsynaptic receptors and excite afferent terminals of the carotid sinus nerve, whose cell bodies reside in the petrosal ganglia (Rong et al., 2003). Others, in contrast, can act as neuromodulators and mediate autocrine or paracrine signalling within the CB to regulate cellular activity (Murali & Nurse, 2016).

The CB afferents make synaptic contacts with compartmentalized second‐order neurons in the caudal portion of the nucleus of the solitary tract in the dorsal medulla oblongata (Zoccal et al., 2014). The CB information is processed within the nucleus of the solitary tract and transmitted to forebrain and brainstem regions that control breathing, the cardiovascular system and metabolism. The neural pathways and transmitters responsible for processing the hypoxia‐induced chemoafferent activity are not fully elucidated, but they include the release of glutamate in the preBötC (Alheid et al., 2011) and the RVLM (Koshiya & Guyenet, 1996).

## 4 CENTRAL O_2_ CHEMORECEPTORS

During conditions of prolonged hypoxia, a progressive and widespread reduction in CNS excitability occurs, in part owing to the accumulation of inhibitory transmitters, such as adenosine (Martin et al., 2007), which contribute to increasing the capacity of the brain to survive during periods of reduced O_2_ supply. However, reductions in O_2_ levels in the brain parenchyma can produce sympathetic excitation and stimulate breathing, as noted during increases in intracranial pressure and reduced cerebral blood flow (the Cushing response) or in experiments using extracorporeal perfusion (Curran et al., 2000; Marina et al., 2020). These observations indicate that the CNS can respond to regional reductions in brain oxygenation not reflected in arterial O_2_ and trigger cardiorespiratory changes during hypoxia independently of the carotid body inputs.

Studies in the 1990s identified that RVLM presympathetic neurons can be activated directly by hypoxia and produce a sympatho‐excitatory response (Sun & Reis, 1994). Later studies reported that the hypoxia‐induced excitatory response of neurons in the ventral medullary surface (including sympathetic and respiratory cells) was retained in the presence of antagonists for glutamate, GABA and glycine receptors (Koganezawa & Paton, 2014). In the CNS, glial cells have the ability to respond to hypoxia and release gliotransmitters, such as ATP (Parkinson et al., 2002). Experiments showing that ATP levels in the ventral surface of the medulla increase during hypoxia (Gourine et al., 2005) suggested that this transmitter could contribute to the excitatory responses to reduced brain O_2_ levels. Later studies identified astrocytes in the ventral medulla, encompassing the respiratory and sympathetic core circuitries, that can sense hypoxia through a mitochondrion‐dependent mechanism that triggers phospholipase C and inositol trisphosphate receptor activation, an increase in intracellular Ca^2+^, and vesicular release of ATP (Angelova et al., 2015) (Figure 1). The ATP released by astrocytes during hypoxia was found to act on purinergic P2Y_1_ receptors expressed on preBötC inspiratory neurons and increase ventilation (Angelova et al., 2015; Rajani et al., 2018). It is still not confirmed whether the RVLM also contains O_2_‐sensitive astrocytes that release ATP to excite sympathetic neurons and cause sympatho‐excitation during central hypoxia. However, this possibility is supported by cell culture studies showing that hypoxia can excite putative C1 presympathetic neurons through a P2Y_1_ receptor‐dependent mechanism (Marina et al., 2015). There are also studies reporting that optogenetic activation of ventromedullary astrocytes can stimulate RVLM neurons via activation of P2Y_1_ receptors (Marina et al., 2013) and that inhibition of vesicular exocytosis in astrocytes residing in the ventral medulla can prevent the sympatho‐excitation in response to reduced cerebral blood flow (Marina et al., 2020).

The idea of central O_2_ sensors is not restricted to the brain. Studies showing that systemic hypoxia can cause responses of sympatho‐excitation and increased blood pressure in spinalysed preparations (Braga et al., 2007; Mathison, 1910) indicate the presence of independent oxygen‐sensitive mechanisms within the spinal cord. Current evidence shows that the intermediolateral cell column, at the thoracic level, contains preganglionic sympathetic neurons that can be excited directly by hypoxia (Barioni et al., 2022) (Figure 1). Their stimulation during low‐O_2_ conditions can generate a sympatho‐excitatory response and initiate gasps (high‐amplitude phrenic bursts) even in the absence of systemic and brainstem hypoxia. Different from the brainstem, this spinal O_2_‐sensing mechanism does not rely on astrocytes but involves crosstalk between neuronal nitric oxide synthase and NADPH oxidase in the preganglionic neurons, with the formation of ROS targeting ionic channels, such as the transient receptor potential channels (Barioni et al., 2022).

It is appealing, therefore, that the brainstem and spinal cord possess sensing mechanisms that can monitor tissue O_2_ levels and promote changes in respiratory and sympathetic activities during central hypoxia. However, it remains to be determined whether other CNS regions can sense central hypoxia and generate appropriate neuronal and functional responses (Erdemli et al., 1998; Horn & Waldrop, 1997).

## 5 THE INTERPLAY OF PERIPHERAL AND CENTRAL O_2_‐SENSING MECHANISMS

Despite peripheral and central O_2_ chemoreceptors displaying comparable sensitivity to hypoxia in vitro (Angelova et al., 2015), their position in different body compartments suggests that these chemosensitive systems are recruited hierarchically during systemic hypoxia. The strategic location of the CB in the carotid artery bifurcation allows the glomus cell to detect reductions in $P_{aO_{2}}$ rapidly and initiate compensatory cardiorespiratory responses to maintain O_2_ homeostasis and adequate tissue oxygenation. However, if O_2_ levels in arterial blood continue to reduce even in the presence of CB‐mediated responses and cause brain hypoxia, the recruitment of central O_2_ chemoreceptors can provide additional excitation to the autonomic and respiratory networks. This possibility is evident in studies showing that brainstem O_2_‐sensitive mechanisms are necessary to support ventilation during prolonged hypoxia, because the antagonism of ATP receptors or inhibition of astrocytes in the ventral medulla potentiates the secondary depression of the HVR (Gourine et al., 2005; Rajani et al., 2018). Therefore, the maintenance of brain integrity during conditions of intense systemic hypoxia might require excitatory inputs from both peripheral and central O_2_‐sensing mechanisms. However, the intricacies of peripheral and central O_2_ chemoreceptor interaction determining the respiratory and autonomic adjustments during different grades of hypoxia require further investigation.

Another perspective on the role of peripheral and central O_2_ sensors relates to the postnatal period. The O_2_ sensitivity of the carotid bodies is low at birth and increases progressively during postnatal development in association with anatomical and functional maturation of the glomus cells (Kholwadwala & Donnelly, 1992). It is not known whether the sensitivity of central O_2_ chemoreceptors changes during postnatal development. However, the sensing and effector mechanisms of the brainstem and spinal cord O_2_‐sensitive cells are functional at birth, with the ability to stimulate the respiratory and sympathetic networks (Angelova et al., 2015; Barioni et al., 2022). Therefore, it is possible to speculate that during exposure to hypoxia at early ages, central O_2_‐sensing mechanisms would be more effective in maintaining brain O_2_ homeostasis and overcoming hypoxia‐induced central depression in the absence of peripheral feedback excitation. During postnatal development, the relative contribution of central and peripheral mechanisms would shift with the increase in CB chemosensitivity. However, these speculations require experimental verification.

## 6 OXYGEN SENSING IN CARDIORESPIRATORY DISEASES

Understanding the functioning of the O_2_ chemoreceptors and their impact on the cardiorespiratory system also has a relevant pathophysiological context. Dysregulation of the O_2_ chemosensory system at the central and peripheral levels can emerge with the progression of some diseases and have additional deleterious effects on the body systems. Likewise, heightened O_2_ chemoreceptor activity can trigger autonomic and cardiorespiratory dysfunctions that impact the quality of life negatively. Therefore, the O_2_ sensors and their signalling pathways have been considered potential targets in therapeutical approaches.

Sleep‐disordered breathing, such as obstructive sleep apnoea (OSA), leads to recurrent hypopnoeas and apnoeas during sleep, episodic hypoxia and stimulation of O_2_ chemoreceptors. The chemoreceptor activation during OSA episodes increases ventilatory efforts to restore normal airflow, causes sympatho‐excitation and haemodynamic changes, and promotes microarousals (Caples et al., 2005). Over time, untreated OSA patients are at the highest risk of developing cardiorespiratory, metabolic and cognitive disorders, such as arterial hypertension, cardiac and vascular diseases, insulin resistance, diabetes mellitus, attention deficits and dementia (Bradley & Floras, 2009; Emamian et al., 2016; Li et al., 2018). Studies in animal models identified that chronic exposure to intermittent hypoxia (CIH) is a relevant risk factor contributing to the negative outcomes of OSA. Clinical and experimental studies indicate that OSA/CIH exposure increases sympathetic activity, enhances the O_2_ sensitivity of CB chemoreceptors and facilitates the ventilatory and sympatho‐excitatory responses to hypoxia (Narkiewicz et al., 1998; Rey et al., 2004; Zoccal et al., 2008). These observations, combined with the fact that CB denervation prevents the development of arterial hypertension (Fletcher et al., 1992), support a crucial role for CIH and the CB‐mediated chemoreflex in the pathophysiology of OSA. The mechanisms underlying the potentiation of CB chemosensitivity by CIH have yet to be elucidated completely. However, compelling evidence indicates that excessive ROS formation in the glomus cells is an important mechanism leading to CB hypersensitivity (Prabhakar et al., 2023). Generation of ROS induced by CIH also occurs in ventromedullary regions encompassing the sympathetic and respiratory core circuitries and central O_2_ sensors (Nanduri et al., 2018), supporting the hypothesis that central alterations are also part of the complex mechanisms underpinning the cardiorespiratory dysfunctions in CIH/OSA.

In children, recurrent episodes of apnoea can occur owing to craniofacial or airway anatomical changes or the immaturity of the respiratory system (Chandrasekar et al., 2022). The latter is common in premature babies, who present an inappropriate neuromuscular control of upper airway patency and failures in the central command of respiratory muscles, leading to periodic obstructive and central apnoeas (Erickson et al., 2021). The breathing instabilities and airway occlusions observed in the apnoea of prematurity or paediatric OSA can expose infants to CIH during a critical period of postnatal development and cause irreversible changes. Augmented hypoxia CB sensory activity has been reported in adult animals exposed to postnatal CIH (Nanduri et al., 2012). It is unclear whether postnatal CIH can alter the functioning of O_2_ central chemoreceptors. However, adult animals that experienced CIH during the postnatal period exhibit increased expression of the transcription factor hypoxia‐inducible factor 1 alpha in the ventrolateral medulla (Karlen‐Amarante et al., 2023), suggesting plasticity in hypoxia‐sensitive mechanisms in the CNS. The sustained alterations in the peripheral and central pathways induced by postnatal CIH contribute to development of arterial hypertension and breathing irregularities in adulthood by epigenetic‐related mechanisms affecting sensory activity and neuronal excitability (Bittencourt‐Silva et al., 2020; Karlen‐Amarante et al., 2023; Nanduri et al., 2012). Additional experiments are required to explore these mechanisms further.

An enhanced CB chemoreflex is also found in patients with chronic heart failure (HF) and animal models of HF. Autonomic dysfunction, especially augmented sympathetic drive to the heart, is a hallmark of HF and is linked directly to disease prognosis and mortality (Franciosi et al., 2017). Moreover, HF patients frequently exhibit an irregular breathing pattern, ranging between periods of fast breathing and apnoeas (Brack et al., 2007). Evidence indicates that increased CB afferent activity mediates, at least in part, the sympathetic and respiratory dysregulation observed in HF (Marcus et al., 2014). The mechanisms underlying the CB hyperactivity in HF are not elucidated fully; however, studies reported that reduced blood flow to the CB and the recruitment of ROS‐related pathways are relevant contributors (Li et al., 2007). These observations have supported clinical trials in which carotid body resection has been performed to reduce sympathetic overdrive in HF patients; a procedure still under debate and consideration (Niewinski et al., 2017).

Arterial hypertension is another relevant and prevalent cardiovascular disease associated with augmented chemoreceptor activity. Clinical and experimental data associate essential hypertension with excessive sympathetic drive and increased CB tonicity and sensory activity (Pijacka et al., 2016; Schlaich et al., 2004). Carotid body denervation in spontaneously hypertensive rats (SHRs) decreased sympathetic activity and blood pressure levels and improved cardiac baroreflex sensitivity, suggesting a causal relationship (Abdala et al., 2012). Similar depressor effects were documented in an animal model of renovascular hypertension after CB removal (Melo et al., 2020). The mechanisms underlying CB hyperactivity in hypertension are poorly understood, but it involves the overexpression of ion channels, such as ASIC3 and TASK (Tan et al., 2010), and an upregulation of P2X_3_‐mediated purinergic signalling in chemosensory petrosal neurons (Pijacka et al., 2016). These experimental findings guided clinical studies exploring new approaches to target CB activity to control arterial blood pressure in patients with resistant hypertension (McBryde et al., 2017). Studies showing that hypertensive patients can exhibit cerebral vertebral hypoplasia and an incomplete posterior circle of Willis associated with a lower cerebral blood flow (Manghat et al., 2022) suggest that impaired cerebral perfusion also contributes to development of high sympathetic drive in arterial hypertension. These observations parallel studies performed in SHRs showing remodelling of the vertebrobasilar arteries and reduced cerebral blood flow in young animals before the onset of hypertension (Cates et al., 2012). Other studies evidenced lower O_2_ levels in the brain parenchyma of SHRs associated with higher hypoxia‐inducible factor 1 alpha expression, astrocyte activation, ATP/lactate release and augmented activity of presympathetic neurons in the ventrolateral medulla (Marina et al., 2015). These findings suggest that reduced cerebral blood flow and central hypoxia are additional mechanisms stimulating the sympathetic network and contributing to the development of arterial hypertension. Whether this central sympatho‐excitatory effect involves the activation of ventromedullary O_2_‐sensitive astrocytes or spinal cord O_2_ sensors remains to be elucidated.

## 7 CONCLUDING REMARKS

The maintenance of O_2_ homeostasis is essential to support cellular activity. The cardiorespiratory system plays a fundamental role in regulating O_2_ uptake and delivery according to the metabolic demands of the body. During systemic hypoxia, ventilatory, haemodynamic and metabolic adjustments emerge to compensate for the lack of O_2_ and maintain cellular integrity, especially in organs with a higher vulnerability, such as the CNS. Peripheral O_2_ sensors in the CBs are an important source of feedback excitation during acute hypoxia, rapidly modifying the activity of respiratory and autonomic networks and their corresponding motor outputs to respiratory muscles, the cardiovascular system and organs controlling the metabolic rate. However, recent studies characterized O_2_ chemoreceptors embedded in the respiratory and sympathetic networks that can monitor tissue O_2_ in the CNS independently of the CBs and evoke respiratory and sympathetic excitatory responses during central hypoxia. Despite the significant discoveries about the functioning of peripheral and central O_2_ chemoreceptors, the clinical evidence showing the involvement of altered hypoxia signalling in the aetiology or worsening of prevalent, sometimes untreatable, cardiorespiratory diseases indicates that there are still unknown pieces in the hypoxia puzzle that need to be unveiled.

## AUTHOR CONTRIBUTIONS

Daniel B. Zoccal conceptualized the review. Daniel B. Zoccal, Beatriz N. Vieira, Letícia R. Mendes, Andressa B. Evangelista and Isabela Paula Leirão provided intellectual input and designed the review. Daniel B. Zoccal and Isabela de Paula Leirão drafted the manuscript. Daniel B. Zoccal, Beatriz N. Vieira, Letícia R. Mendes, Andressa B. Evangelista and Isabela de Paula Leirão corrected and approved the final version of the manuscript.

## CONFLICT OF INTEREST

Data sharing is not applicable to this article as no new data were created or analyzed in this study.
